# Dataset of building locations in Poland in the 1970s and 1980s

**DOI:** 10.1038/s41597-024-03179-2

**Published:** 2024-04-05

**Authors:** Piotr Szubert, Dominik Kaim, Jacek Kozak

**Affiliations:** 1 Jagiellonian University, Doctoral School of Exact and Natural Sciences, Prof. St. Łojasiewicza St 11, 30-348 Cracow, Poland; 2 Institute of Geography and Spatial Management, Faculty of Geography and Geology, Jagiellonian University, Gronostajowa 7, 30-387 Cracow, Poland

**Keywords:** Environmental impact, Sustainability

## Abstract

The aim of this study was to create a dataset of building locations in Poland from the 1970s–1980s. The source information was the historical 1:10 000 Polish topographic map. Building footprints were detected and extracted from approximately 8,500 scanned map sheets using the Mask R-CNN model implemented in Esri ArcGIS Pro software, and converted to point building locations. The dataset of building locations covers the entire country and contains approximately 11 million points representing buildings. The accuracy of the dataset was assessed manually on randomly selected map sheets. The overall accuracy is 95% (F1 = 0.98). The dataset may be used in conjunction with various contemporary land use, land cover and cadastral datasets in a broad range of applications related to long-term changes in rural and urban areas, including urban sprawl and its environmental and social consequences. It can also serve as a highly reliable reference dataset for regional or global settlement products derived, e.g., from early Landsat data.

## Background & Summary

### Background

Built-up areas and individual buildings are currently mapped with high accuracy using Earth observation (EO) data and machine learning methods^1^. At the global scale, built-up areas are included in land cover datasets such as a WorldCover dataset based on Sentinel-2 image data (10 m)^2^ and the GLC_FCS30 dataset based on Landsat image data (30 m)^3^. The World Settlements Footprint Evolution dataset^4^, based on Landsat mission image data, provides built-up area coverage from 1985 to 2015 at a 30 m resolution. Other global scale datasets are Global Urban Footprint (GUF) datasets^5^ with a resolution of 0.4” based on radar EO missions and Global Artificial Impervious Areas (GAIA) datasets^6^ covering changes in impervious areas from 1985 to 2018. The Global Human Settlement Layer (GHSL)^7^ of the European Joint Research Centre is an example of a dataset that combines EO products with census data. At the continental scale, the European Copernicus programme provides various accurate, high-resolution data on built-up area distributions derived from EO data, so-called Imperviousness High Resolution Layers or, quite recently, CORINE+ data with 10 m spatial resolution^8^. In addition, many countries maintain real-time registers of existing buildings and new buildings being constructed or removed; these records are then available as spatial data with high accuracy and detail. These datasets include, for instance, Austrian Cadaster (Österreichischer Kataster) in Austria^9^ and Evidence of Grounds and Buildings (Ewidencja Gruntów i Budynków) in Poland^10^. The detailed cadastral data then feed modern digital topographic open datasets containing building-level spatial information at the national level, such as the British Ordnance Survey Master Map or Polish BDOT10K. Furthermore, major global IT companies develop their own datasets, which are frequently made openly available to the public. Microsoft’s Bing Maps published an accurate global building footprints dataset under the Open Data Commons Open Database License^11^. The Google Maps team has published their own building dataset created with a deep learning model for Africa, Latin America and Southeast Asia^12^, while Meta has developed the Data for Good dataset^13^. Apart from official data sources, other open source datasets, such as OpenStreetMap, provide users with high-quality information about building locations^14–16^.

However, historical information on buildings’ locations is rarely available for large areas except in the form of paper topographic maps or cadastral data. Even more generalized, settlement pattern maps of large areas have been available only since the 1970s, and their quality for the oldest editions is substantially lower than that for contemporary products^17,18^. Uhl and Leyk overcome this issue by developing methods based on real estate databases^19^ and combining modern buildings footprints datasets with historical tax and parcels datasets^20^, significantly extending the time range of their datasets. However, these methods entail greater uncertainty in the resulting datasets, while official cartographical materials present geographical data with high accuracy and precision. Historical map datasets are frequently used as data sources in various research fields, e.g., land cover change studies^21–28^ and archaeology^29^. However, the sizes of case studies and the thematic scope of related research are limited by data availability^28^. Therefore, there is a need to collect information from archival datasets and make them easily available to researchers^24^.

One of the main challenges in extracting information from old maps is the complexity of cartographic presentation; therefore, researchers frequently rely on manual delimitation of features^27^. Over time, various methods for extracting information from paper maps have been proposed, for instance, methods based on color segmentation^23,24,27,30,31^, combining multiple source datasets^19,20,32^ or crowdsourcing feature extraction among volunteers^33^. Recently, machine learning methods, in particular those based on deep learning, have become the state of the art in image recognition, providing high-quality outputs and allowing the processing of much larger datasets than older methods. Several authors have successfully tested the ability of these methods to extract features from archival maps, such as spot elevation marks^34^, geographical object labels^35^, wetlands^36,37^, roads^38^, archaeological sites^29^, cadastral information^39^, and buildings and settlements^40,41^. To date, however, large-scale databases on historical building locations are still rare or cover relatively small areas^42^.

With respect to land use and land cover change, Poland is an interesting case study due to the rapid development in rural and urban areas after the collapse of socialism in the late 1980s, the market-oriented transformation in the 1990s and the relatively weak restrictions enforced by spatial planning laws that have not prevented uncontrolled built-up area sprawl, with various environmental and societal consequences^43^. To study transformation-related changes in building locations, distribution and density across the entire country, relevant data need to be massively extracted from existing paper maps, representing building locations prior to the transformation, that is, in the 1970s and 1980s.

### Objectives of the paper and summary

**Fig. 1:**
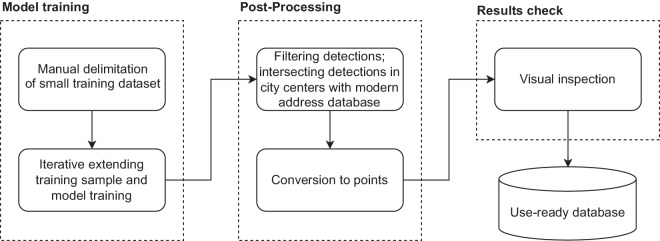
Simplified workflow diagram.

In this paper, we introduce a dataset of buildings covering all of Poland (312,000 km^2^) for the 1970s and 1980s. To achieve this goal, we developed a method of building location extraction from a consistent edition of topographic paper maps using the Mask R-CNN deep learning model^44^ (Fig. 1) implemented in the ESRI ArcGIS Pro software.

**Fig. 2:**
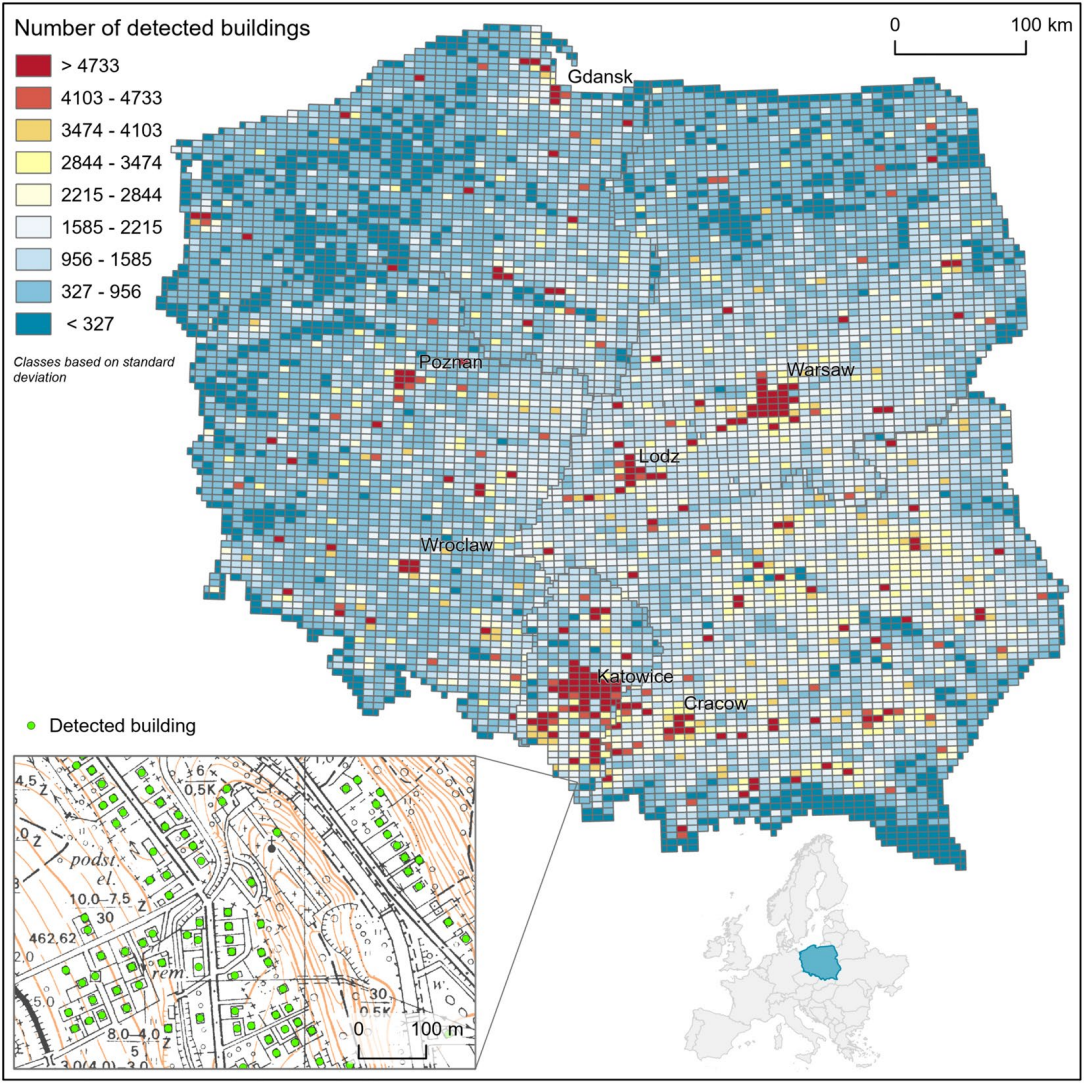
Building locations in Poland in 1970s and 1980s.

The dataset covers 10,988,583 buildings detected on official topographic maps at the scale of 1:10 000 (Fig. 2). It is the only countrywide digital dataset presenting this historical information, which may be easily compared to contemporary topographic data representing building locations in the 21^st^ century. Therefore, the dataset may be a main source of information for various socioenvironmental studies focusing on long-term changes in urban and rural settings in Poland. It can also be used as a highly reliable reference dataset for regional or global scale settlement reconstructions involving the period that the analyzed maps depict. Future works could focus on expanding the building location dataset with additional attributes, e.g., building area and usage, extracted either from the source historical maps or received through integration of the historical building location data with various contemporary datasets.

## Methods

### Materials

**Fig. 3:**
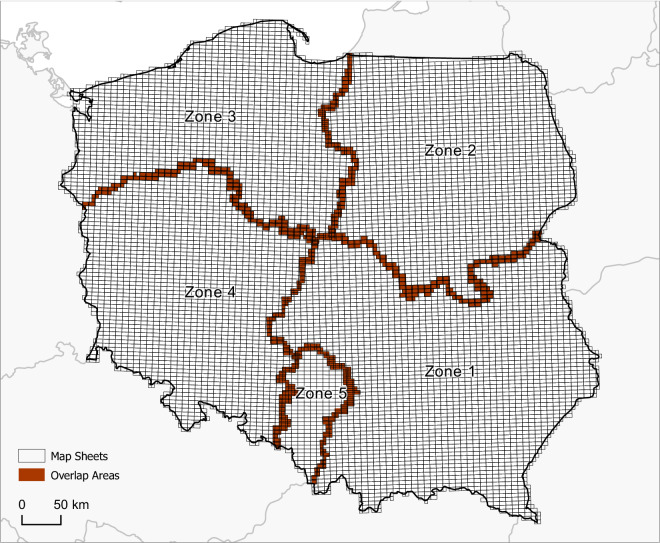
Map sheets coverage extent. Data from the Office of Geodesy and Cartography, Poland.

The basic data source of the study included 8579 historical topographic map sheets at a scale of 1:10 000 covering the entire of Poland (Fig. 3). The dataset was provided by Główny Urząd Geodezji i Kartografii (GUGiK, Head Office of Geodesy and Cartography), the National Government Surveying and Cartography Agency^45^. All map sheets are made available by GUGiK as scans in geotiff format with 0.5 m spatial resolution, georeferenced and transformed from their original coordinate system (“1965”) to the currently used “PL-1992” (EPSG:2180) coordinate system.

**Fig. 4:**
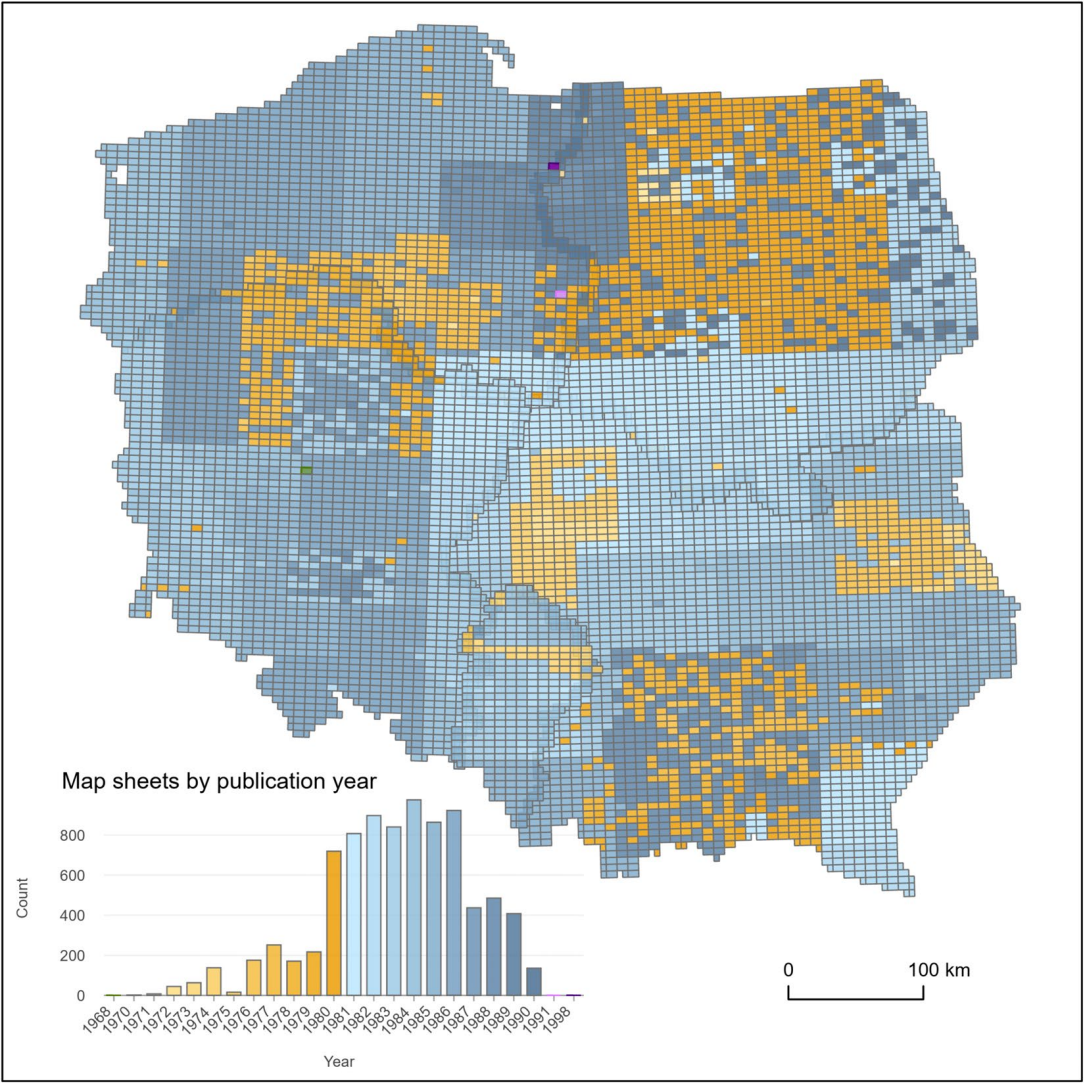
Map sheets publication years. Data from the Office of Geodesy and Cartography, Poland.

**Fig. 5:**
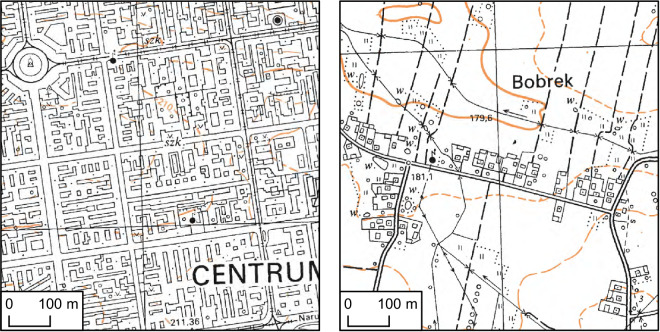
Source topographic maps. Example of city center (left) and village (right). Data from the Office of Geodesy and Cartography, Poland.

The maps were produced from the late 1960s until the 1990s (Fig. 4). The vast majority of the map sheets (Fig. 4) were published between 1980 and 1986, and almost the entire set was published between 1976 and 1989 (88%). This was the first and only civil edition of topographic maps that covered the whole country in such detail (scale 1:10 000) in the entire postwar period up to the beginning of the 21^st^ century^46^. As a result of the “1965” coordinate system definition, overlap areas between different zones of the system (Fig. 3) were mapped independently for each zone of the overlap. Areas outside the national boundary were masked. The map prints are bicolour, with contours shown in brown/orange and all other signatures shown in black. All signatures of areal objects are represented as white polygons with black outlines. The shapes of building footprints differ in various regions and depend on the type of settlement. In rural areas, most buildings are represented by rectangles. Similarly to buildings in densely built-up areas, large industrial buildings were represented by more complex polygons, for instance, in historical centers of old cities (Fig. 5). The other types of black color signatures are linear features, representing riverbanks, all types of roads, railway tracks, embankments, utility infrastructure and other objects.

To fine-tune the results of building extraction, we also used the current national address base dataset included in the National Boundaries Register (Państwowy Rejestr Granic). The dataset is available freely through the Polish Geoportal^47^.

## Methods

The delimitation of building footprints was based on the Mask R-CNN deep learning model^44^ implemented in Esri ArcGIS Pro software^48^. The model was designed to perform instance segmentation, object detection and classification. For each detected building, the model provides a polygon mask of the building footprint and a confidence factor. Then, the model detections were converted to points, postprocessed and uploaded to the database. The approach consisted of four major stages: model training, model execution, postprocessing and validation.

### Model training

**Fig. 6:**
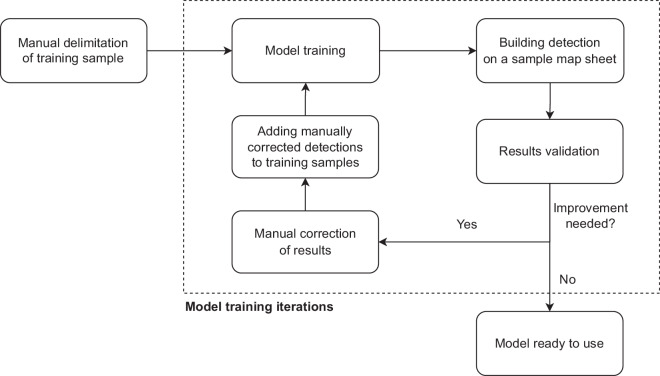
Model training process.

To detect building footprints, the model was trained on a representative training sample. To avoid manual delimitation of thousands of buildings, an iterative approach^40^ was used instead, with limited manual input. After training the model on a small initial training sample from a selected map sheet, the whole map sheet was classified with a model, and the results were manually corrected and added to the training sample used in the next iteration. The final training sample was obtained in three iterations (Fig. 6).

In the first iteration, the manually collected training sample contained polygons of 200 building footprints represented as raster masks. They were delimited from map sheet no. 184.113, covering the area south of Nowy Sącz, a middle-sized town in the mountainous part of the country. The area was chosen because it contains various building types (rural, urban, peri-urban and industrial facilities) with different densities of contours intersecting their signatures. The first model was trained for 10 epochs. The model performance during training was evaluated with respect to the loss function values in the training and validation datasets, which is common practice in deep learning^49^. The first model achieved 0.52 training loss and 0.58 validation loss. We used the trained model to detect buildings on the entire ‘184.113’ map sheet. The results were then manually corrected, false positive detections were deleted, and missing buildings were delimited and added to the sample, which contained approximately 6000 buildings.

In the second iteration, the model was trained with 10 epochs, achieving 0.49 training loss and 0.54 validation loss, using two map sheets covering different types of settlements. For training, we used sheet ‘186.311’ covering rural mountain areas in the Eastern Carpathians with dispersed farm buildings and sheet no. 165.344, covering the city of Rzeszów, with a variety of different types of buildings, such as industrial halls, residential blocks, detached houses and high-density city housing. Again, the results were manually corrected and added to the training data, resulting in a training sample of approximately 16,000 building footprints.

In the third iteration, the model was trained with 10 epochs, achieving 0.49 training loss and 0.54 validation loss using four map sheets located in various regions in Poland (map sheets no. 131.434, 144.441, 214.313 and 223.342). The results of the third iteration were visually evaluated as satisfactory. Approximately 9,500 buildings were detected and added to the training sample. As further extension of the training sample did not improve the model performance, the model achieved after the third iteration was used in further work.

### Model execution

Maps were processed using Python script based mostly on Esri’s arcpy library. The average processing time of one map sheet was approximately 15 minutes using a computer with a GeForce Gtx 1080 Ti graphics card. To fit the maps to the model input size, the map sheets were split into 256 × 256 pixel overlapping tiles, with an overlap of 56 pixels. The model was executed independently on each tile using the ‘Detect Objects Using Deep Learning’ arcpy function in the script. The detected objects were then vectorized and stored in separate files for each map sheet. Duplicate detections from the edges of overlapping tiles were kept as distinct detections at this stage.

### Postprocessing

#### Finding a threshold to filter low-quality detections

**Fig. 7:**
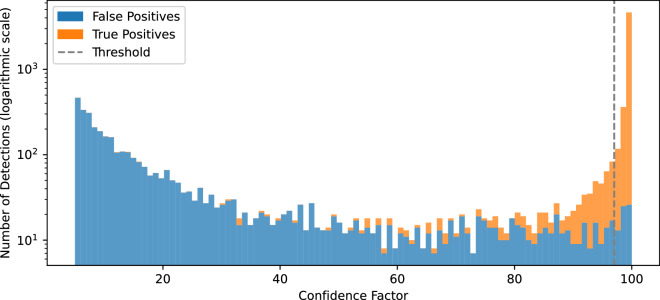
Detected building: distribution of the confidence factor.

We filtered results with confidence factors to remove most of the false positive results. The threshold value was set up based on the validation of the sheets used in the third iteration of training (map sheets no. 131.434, 144.441, 214.313 and 223.342), covering different types of settlements. Based on the histogram of false positives and true positives, the threshold was set at 0.97 (Fig. 7), allowing us to remove most of the false positives without removing too many true positives.

#### Building footprints to points

The model outputs were polygons representing building detections with their individual confidence values. To eliminate duplications of the same building in the overlaps of the 256 × 256 tiles, the resulting polygon features were merged based on their location, assigning the highest confidence value among overlapping detections to the merged polygon representing a detected building. Then, all polygons were converted into points with the feature-to-point tool from Esri’s arcpy library. The tool was set up to create points inside the polygons.

#### Building extraction in historical city centers

**Fig. 8:**
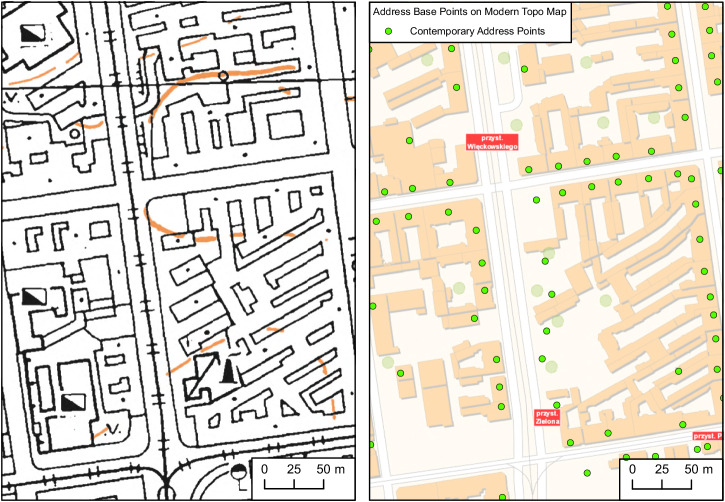
Separation of buildings in city centers. Data from the Office of Geodesy and Cartography, Poland.

**Fig. 9:**
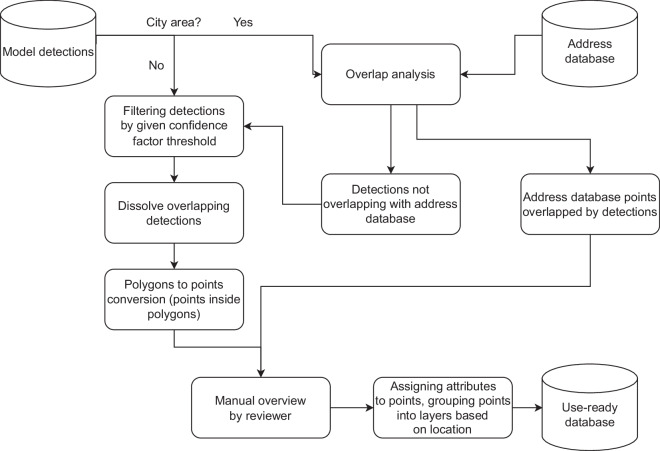
Postprocessing of extracted buildings in the historical city centers.

As whole quarters in densely built-up historical centers were represented on the maps using a single polygon (Fig. 5), it was not possible to extract locations of single buildings belonging to the quarter. In this context, a single building was understood as a part of a larger structure having a unique postal address (Fig. 8). 507 map sheets were manually classified as containing dense built-up areas. Footprints detected from these maps were intersected with the modern address database (Fig. 9). If an address point was contained in a building footprint, then it was added to the database as a point representing a building location. Historical city centers have not experienced rapid changes since the time of source map creation. Therefore, modern address points are unlikely to refer to buildings other than those represented on historical maps as consolidated building footprints.

#### Visual inspection

**Fig. 10:**
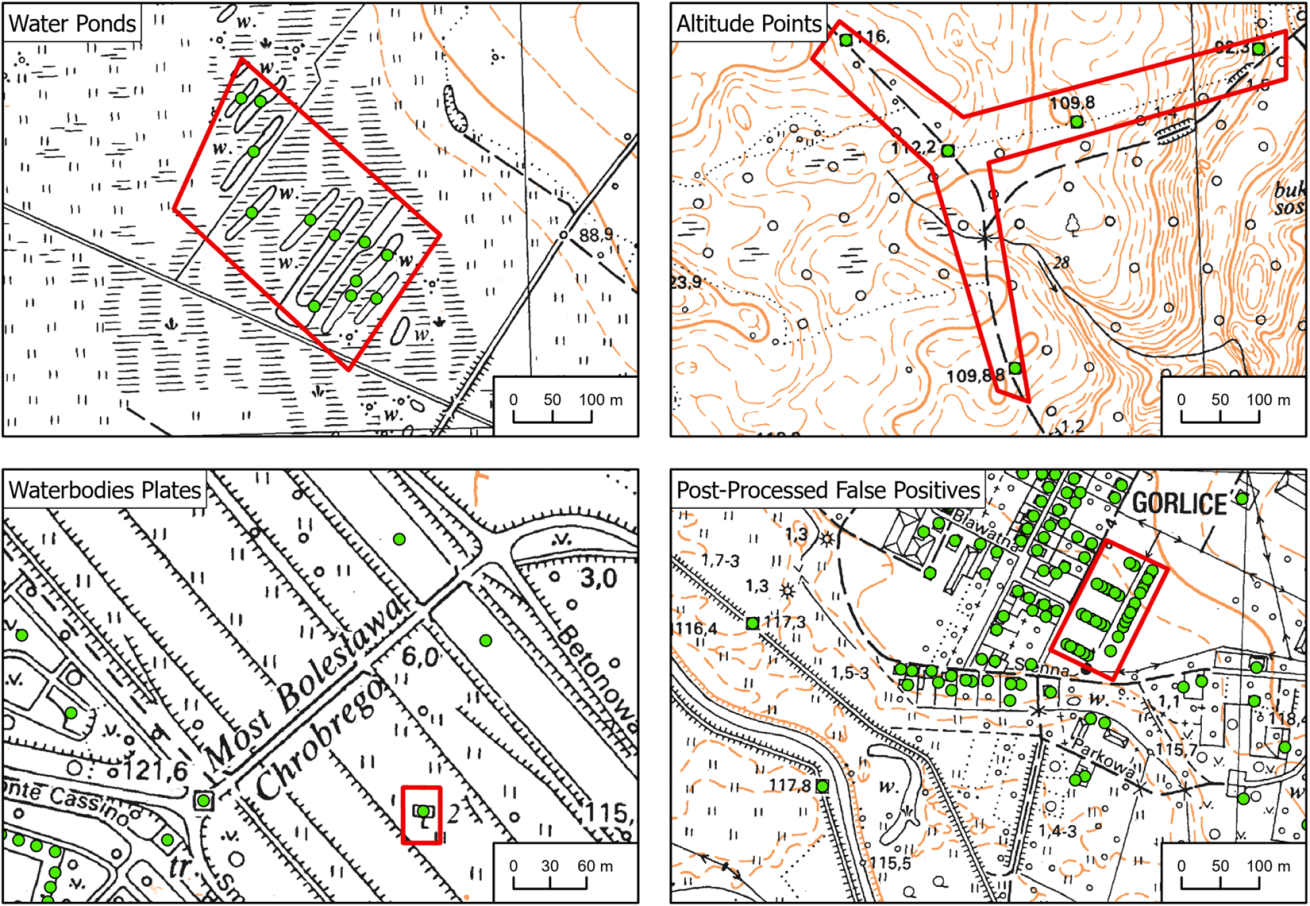
False positive detections after postprocessing and before visual inspection. Data from the Office of Geodesy and Cartography, Poland.

To remove repetitive false-positive detections (Fig. 10), each map sheet was visually inspected and, if necessary, manually corrected. The main goal was to delete the most common false positives, such as altitude marks and road numbers. The average duration of manual correction of one map sheet was approximately 3 minutes.

#### Assigning attributes to points and splitting points into layers

Finally, each point representing the building location received information about the map sheet number and map production year, which was stored in the attribute table. Points in overlapping areas of the coordinate system zones and points imported from the address base were marked with Boolean flags. The buildings were finally organized in database tables based on the current administrative division (NUTS-2 level - voivodships).

## Data Records

The database is available at the Zenodo service (10.5281/zenodo.8373083)^50^. The data is divided into tables based on the current administrative divisions in Poland. Each table contains the following fields: Tile, which contains map sheet number; Year, which stores the map production year; AddressBase, which includes true values assigned to points imported from the address database; and Overlapping, which flags points from coordinate system zones overlapping areas. All the data is stored in the WGS84 coordinate system (EPSG:4326).

The database is shared in the open-source GeoPackage format. It can be used in any commercial or freely available GIS software supporting SQL Lite databases. It can also be accessed through database interfaces such as the DB Browser.

## Technical Validation

**Fig. 11:**
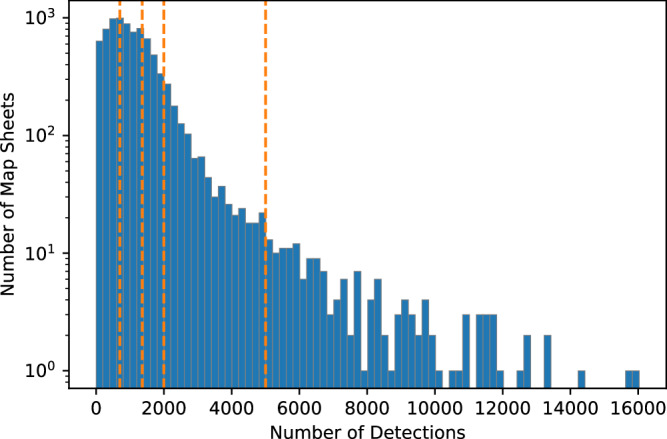
Distribution of detected buildings per map sheet.

The results were manually validated using a stratified sampling method. We divided all map sheets into 5 subgroups based on the number of detected buildings (Fig. 11): 0–700; 700–1360; 1360–2000; 2000–5000, >5000. From each group, we randomly chose 5 map sheets. The chosen map sheets contained in total 69,961 detected buildings and included all types of settlements in rural and urban areas located in various landscapes. In the assessment, we manually identified correctly detected buildings, false positives and false negatives.

**Fig. 12:**
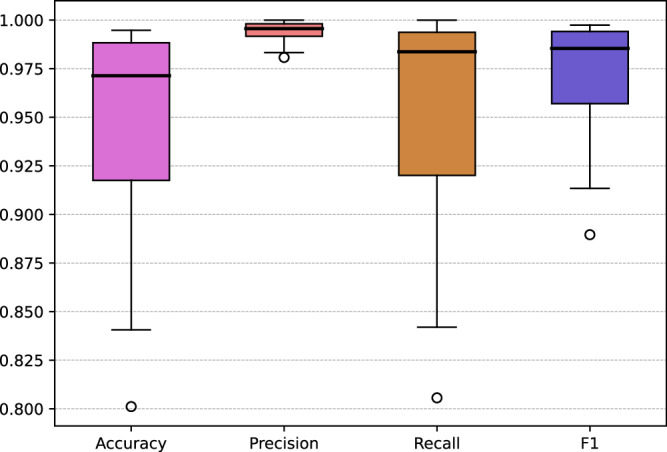
Performance metrics distribution in the validation set.

**Fig. 13:**
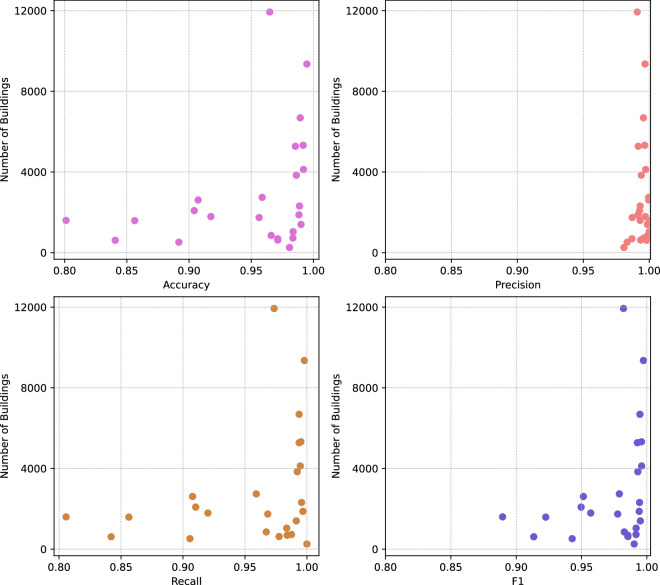
Performance metrics related to the number of buildings on map sheets used in validation.

**Fig. 14:**
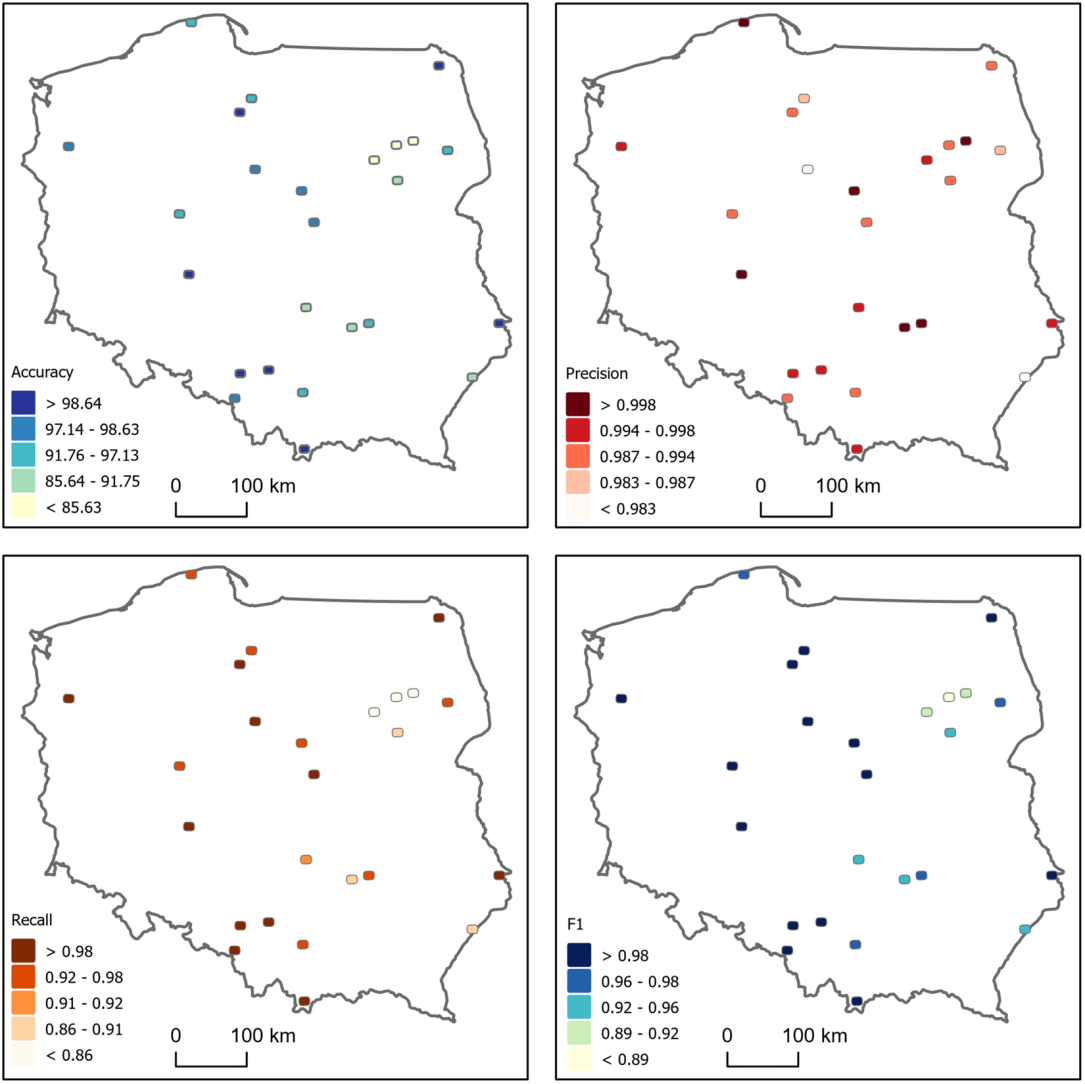
Maps of performance metrics.

We found a true positive rate of 0.99, a false positive rate of 0.01 and a false negative rate of 0.03. The median accuracy, median F1 score, median precision and mean recall were 0.97, 0.99, 0.99 and 0.98, respectively (Fig. 12), with most validation sets presenting highly accurate results (Fig. 12). The validation map sheets with fewer buildings are more diverse in terms of the overall accuracy, recall and F1 metric results (Fig. 13). Validation map sheets with the lowest overall accuracy, Recall and F1 are located in the northeastern part of the country in the area of lowland meandering rivers valleys (Fig. 14). False positive detections in that area were related to small water pounds and unique rural settlements structures (Fig. 10).

## Data Availability

Data processing was performed using the ESRI Arcpy Python Library. Spatial analysis and map production was done using ArcGIS Pro software. Plots were generated with Matplotlib Python Library. Code written for maps processing and detections postprocessing is available at GitHub repository: https://github.com/Szubbi/WallToWallMapingBuildingsPoland.
